# COVID-19 vaccine-induced antibody and T-cell responses in immunosuppressed patients with inflammatory bowel disease after the third vaccine dose (VIP): a multicentre, prospective, case-control study

**DOI:** 10.1016/S2468-1253(22)00274-6

**Published:** 2022-09-09

**Authors:** James L Alexander, Zhigang Liu, Diana Muñoz Sandoval, Catherine Reynolds, Hajir Ibraheim, Sulak Anandabaskaran, Aamir Saifuddin, Rocio Castro Seoane, Nikhil Anand, Rachel Nice, Claire Bewshea, Andrea D'Mello, Laura Constable, Gareth R Jones, Sharmili Balarajah, Francesca Fiorentino, Shaji Sebastian, Peter M Irving, Lucy C Hicks, Horace R T Williams, Alexandra J Kent, Rachel Linger, Miles Parkes, Klaartje Kok, Kamal V Patel, Julian P Teare, Daniel M Altmann, James R Goodhand, Ailsa L Hart, Charlie W Lees, Rosemary J Boyton, Nicholas A Kennedy, Tariq Ahmad, Nick Powell, VIP study investigators, Ijeoma Chukwurah, Sulaimaan Haq, Parita Shah, Stephanie Wilken-Smith, Anitha Ramanathan, Mikin Patel, Lidia Romanczuk, Rebecca King, Jason Domingo, Djamila Shamtally, Vivien Mendoza, Joanne Sanchez, Hannah Stark, Bridget Knight, Louise Bee, Charmaine Estember, Anna Barnes, Darcy Watkins, Sam Stone, John Kirkwood, Marian Parkinson, Helen Gardner-Thorpe, Kate Covil, Lauranne Derikx, Beatriz Gros Alcalde, Irish Lee, Bessie Cipriano, Giuseppe Ruocco, Manisha Baden, Graham Cooke, Katrina Pollock, Evgenia Kourampa, Ciro Pasquale, Elena Robisco-Diaz, Suhaylah Bhatti

**Affiliations:** a Department of Metabolism, Digestion and Reproduction, Imperial College London, London, UK; b Department of Infectious Disease, Imperial College London, London, UK; c Department of Surgery and Cancer, Imperial College London, London, UK; d Department of Immunology and Inflammation, Imperial College London, London, UK; e Department of Gastroenterology, Imperial College Healthcare NHS Trust, London, UK; f Division of Medicine and Integrated Care, Imperial College Healthcare NHS Trust, London, UK; g Department of Gastroenterology, St Mark's Hospital and Academic Institute, London, UK; h Exeter Inflammatory Bowel Disease and Pharmacogenetics Research Group, University of Exeter, Exeter, UK; i Department of Clinical Chemistry, Biochemistry, Exeter Clinical Laboratory International, Royal Devon and Exeter NHS Foundation Trust, Exeter, UK; j Department of Gastroenterology, Royal Devon and Exeter NHS Foundation Trust, Exeter, UK; k Department of Gastroenterology, Western General Hospital, NHS Lothian, Edinburgh, UK; l Centre for Inflammation Research, The Queen's Medical Research Institute, The University of Edinburgh, Edinburgh, UK; m Nightingale-Saunders Clinical Trials and Epidemiology Unit, King's Clinical Trials Unit, King's College London, London, UK; n School of Immunology and Microbial Sciences, King's College London, London, UK; o Department of Gastroenterology, Hull University Teaching Hospitals NHS Trust, Hull, UK; p Hull York Medical School, University of Hull, Hull, UK; q Department of Gastroenterology, Guy's and St Thomas' NHS Foundation Trust, London, UK; r Lung Division, Royal Brompton and Harefield Hospitals, Guy's and St Thomas' NHS Foundation Trust, London, UK; s Department of Gastroenterology, King's College Hospital, London, UK; t The NIHR Bioresource, University of Cambridge, Cambridge, UK; u Department of Gastroenterology, Cambridge University Hospitals NHS Trust, Cambridge, UK; v Department of Gastroenterology, Bart's Health NHS Trust, London, UK; w Department of Gastroenterology, St George's Hospital NHS Trust, London, UK

## Abstract

**Background:**

COVID-19 vaccine-induced antibody responses are reduced in patients with inflammatory bowel disease (IBD) taking anti-TNF or tofacitinib after two vaccine doses. We sought to assess whether immunosuppressive treatments were associated with reduced antibody and T-cell responses in patients with IBD after a third vaccine dose.

**Methods:**

VIP was a multicentre, prospective, case-control study done in nine centres in the UK. We recruited immunosuppressed patients with IBD and non-immunosuppressed healthy individuals. All participants were aged 18 years or older. The healthy control group had no diagnosis of IBD and no current treatment with systemic immunosuppressive therapy for any other indication. The immunosuppressed patients with IBD had an established diagnosis of Crohn's disease, ulcerative colitis, or unclassified IBD using standard definitions of IBD, and were receiving established treatment with one of six immunosuppressive regimens for at least 12 weeks at the time of first dose of SARS-CoV-2 vaccination. All participants had to have received three doses of an approved COVID-19 vaccine. SARS-CoV-2 spike antibody binding and T-cell responses were measured in all participant groups. The primary outcome was anti-SARS-CoV-2 spike (S1 receptor binding domain [RBD]) antibody concentration 28–49 days after the third vaccine dose, adjusted by age, homologous versus heterologous vaccine schedule, and previous SARS-CoV-2 infection. The primary outcome was assessed in all participants with available data.

**Findings:**

Between Oct 18, 2021, and March 29, 2022, 352 participants were included in the study (thiopurine n=65, infliximab n=46, thiopurine plus infliximab combination therapy n=49, ustekinumab n=44, vedolizumab n=50, tofacitinib n=26, and healthy controls n=72). Geometric mean anti-SARS-CoV-2 S1 RBD antibody concentrations increased in all groups following a third vaccine dose, but were significantly lower in patients treated with infliximab (2736·8 U/mL [geometric SD 4·3]; p<0·0001), infliximab plus thiopurine (1818·3 U/mL [6·7]; p<0·0001), and tofacitinib (8071·5 U/mL [3·1]; p=0·0018) compared with the healthy control group (16 774·2 U/mL [2·6]). There were no significant differences in anti-SARS-CoV-2 S1 RBD antibody concentrations between the healthy control group and patients treated with thiopurine (12 019·7 U/mL [2·2]; p=0·099), ustekinumab (11 089·3 U/mL [2·8]; p=0·060), or vedolizumab (13 564·9 U/mL [2·4]; p=0·27). In multivariable modelling, lower anti-SARS-CoV-2 S1 RBD antibody concentrations were independently associated with infliximab (geometric mean ratio 0·15 [95% CI 0·11–0·21]; p<0·0001), tofacitinib (0·52 [CI 0·31–0·87]; p=0·012), and thiopurine (0·69 [0·51–0·95]; p=0·021), but not with ustekinumab (0·64 [0·39–1·06]; p=0·083), or vedolizumab (0·84 [0·54–1·30]; p=0·43). Previous SARS-CoV-2 infection (1·58 [1·22–2·05]; p=0·0006) was independently associated with higher anti-SARS-CoV-2 S1 RBD antibody concentrations and older age (0·88 [0·80–0·97]; p=0·0073) was independently associated with lower anti-SARS-CoV-2 S1 RBD antibody concentrations. Antigen-specific T-cell responses were similar in all groups, except for recipients of tofacitinib without evidence of previous infection, where T-cell responses were significantly reduced relative to healthy controls (p=0·021).

**Interpretation:**

A third dose of COVID-19 vaccine induced a boost in antibody binding in immunosuppressed patients with IBD, but these responses were reduced in patients taking infliximab, infliximab plus thiopurine, and tofacitinib. Tofacitinib was also associated with reduced T-cell responses. These findings support continued prioritisation of immunosuppressed groups for further vaccine booster dosing, particularly patients on anti-TNF and JAK inhibitors.

**Funding:**

Pfizer.

**Research in context**

**Evidence before this study**

We searched PubMed and Embase, without language restrictions, for studies published between Jan 1, 2000, and Jul 31, 2022, investigating humoral or T cell responses to vaccination in immunosuppressed individuals. We used the search terms (“vaccine” OR “vaccination”) AND (“immunosuppression” OR “immunosuppressive” OR “immunomodulator” OR “thiopurine” OR “azathioprine” OR “biologic” OR “tumour necrosis factor” OR “infliximab” OR “ustekinumab” OR “anti-integrin” OR “vedolizumab” OR “JAK inhibitor” OR “tofacitinib”) AND (“antibody” OR “humoral” OR “immune response”) OR (“T cell”). We have previously shown diminished COVID-19 vaccine-induced antibody responses in patients with inflammatory bowel disease (IBD) taking infliximab and tofacitinib, but not vedolizumab or thiopurine monotherapy, following two vaccine doses. Multiple studies have shown that anti-TNF treatment is associated with lower antibody responses, and CLARITY-IBD found no difference in T-cell responses between patients treated with infliximab and those treated with vedolizumab following a second vaccine dose. Breakthrough infection is more common in patients with IBD receiving infliximab compared with vedolizumab after two vaccine doses. There are scarce data on humoral and cell-mediated anti-SARS-CoV-2 immunity in patients with IBD compared with non-immunosuppressed healthy control groups after three COVID-19 vaccine doses.

**Added value of this study**

To our knowledge, this is the first study to evaluate humoral and cell-mediated immune responses following three doses of COVID-19 vaccine in patients receiving different immunosuppressive treatments used in IBD. We showed that, although all groups had a significant boost in vaccine-induced anti-SARS-CoV-2 spike antibody binding after a third dose, levels were significantly reduced in those patients treated with infliximab or tofacitinib. Tofacitinib recipients also had significantly reduced T-cell responses against spike protein compared with the healthy control group.

**Implications of all the available evidence**

In line with other studies, our data show that a third dose of COVID-19 vaccine boosts anti-SARS-CoV-2 spike (S1 receptor binding domain) antibody binding irrespective of immunosuppressive treatment. Combined with evidence that previous SARS-CoV-2 infection further augments humoral responses to vaccination, these results support the roll out of booster doses in immunosuppressed patients with IBD. In the context of emerging variants of concern, and evidence that patients treated with anti-TNF are at higher risk of breakthrough infection, our data also support the prioritisation of future booster dosing to those with diminished responses to vaccination, including patients taking anti-TNF or tofacitinib.

## Introduction

The COVID-19 pandemic has accounted for more than 6 million deaths worldwide as of July, 2022.^1^ Vaccination has been the most effective means of reducing hospitalisations and deaths.^2^, ^3^ Several vaccines have now been approved, including mRNA, adenovirus vector, and protein-based platforms.^4^ However, because patients with immune mediated inflammatory disorders such as inflammatory bowel disease (IBD) were excluded from vaccine trials, data on the efficacy of vaccines in these patients are scarce. The VIP study is a prospective, multicentre study seeking to assess whether COVID-19 vaccine immunogenicity is altered in patients receiving the commonly prescribed immunosuppressive treatments. Previously, we reported that patients with IBD taking the anti-TNF treatment infliximab or the JAK inhibitor tofacitinib had significantly reduced anti-SARS-CoV-2 spike antibody binding compared with healthy controls after two doses of vaccine.^5^ Other commonly used immunosuppressants, including thiopurines, ustekinumab, and vedolizumab, were not associated with a reduction in antibody binding. Evidence is emerging that antibody concentrations decrease more rapidly in patients with IBD treated with anti-TNF drugs and that these patients are at greater risk of breakthrough infection following two doses of vaccine than patients with IBD treated with vedolizumab.^6^, ^7^, ^8^

In some countries, including the UK, immunosuppressed patients have been prioritised for third primary doses and booster doses of vaccine,^9^ and in the UK, uptake of third doses amongst immunosuppressed patients with IBD has been reported at 79%.^10^ Data about immunity following third vaccine doses in patients with IBD are limited, and interpretation is problematic due to a lack of healthy control participants or data about cell-mediated immunity.^11^ We have shown that a two-dose schedule of mRNA vaccine is associated with higher anti-SARS-CoV-2 spike antibody binding than two doses of adenovirus vector vaccine in the immunosuppressed population with IBD.^5^ Although in North America homologous mRNA vaccine schedules have been used almost exclusively, in the UK and worldwide, heterologous vaccination schedules (eg, two doses of adenovirus vaccine followed by one dose of mRNA vaccine) have been used. Heterologous boosting is effective in healthy individuals;^12^ however, further research is needed in immunosuppressed individuals. Finally, although antibody responses to SARS-CoV-2 vaccination in patients with IBD have been the subject of a growing body of research,^13^, ^14^, ^15^, ^16^, ^17^ there is a scarcity of data on the effect of immunosuppressive therapies on T-cell immunity after vaccination in this setting.^6^, ^18^

In this study, we investigated antibody and T-cell-mediated immunity against the SARS-CoV-2 spike protein following three doses of vaccine in patients with IBD who were taking commonly prescribed immunosuppressive treatments.

## Methods

### Study design and participants

VIP was a multicentre, prospective, case-control study done in nine hospital centres in the UK.^5^

We recruited immunosuppressed patients with IBD and non-immunosuppressed healthy individuals. All participants were aged 18 years or older. The inclusion criteria for the healthy control group were no diagnosis of IBD and no current treatment with systemic immunosuppressive therapy for any other indication. Healthy controls were not excluded if they had other medical conditions. The healthy control group was recruited from healthy volunteer databases and from staff working at medical and university centres involved in the study. Inclusion criteria for six groups of immunosuppressed patients with IBD were an established diagnosis of Crohn's disease, ulcerative colitis, or unclassified IBD using standard definitions of IBD, and established treatment with one of six immunosuppressive regimens (thiopurine, infliximab monotherapy, infliximab and thiopurine combination therapy, ustekinumab monotherapy, vedolizumab monotherapy, or tofacitinib monotherapy) for at least 12 weeks at the time of first dose of SARS-CoV-2 vaccination. Exclusion criteria were treatment with any other immunosuppressive treatments or treatment combinations including methotrexate, adalimumab, or ciclosporin. Current treatment with systemic corticosteroids was not an exclusion criterion. The full study protocol can be viewed online. Participants were included after providing informed, written consent.

To be included in this analysis of the VIP cohort, participants had to have received three doses of an approved COVID-19 vaccine. Participants either received a homologous vaccination schedule (three doses of an mRNA vaccine) or a heterologous vaccine schedule (two doses of adenovirus vector vaccine followed by a dose of an mRNA vaccine).

The Wales Research Ethics Committee 5 approved the study (REC reference 21/WA/0105) in March, 2021.

### Procedures

Laboratory analysis of serology was done at the Academic Department of Blood Sciences at the Royal Devon and Exeter NHS Foundation Trust. To determine vaccine specific antibody responses the Elecsys Anti-SARS-CoV-2 S (Roche, Rotkreuz, Switzerland) was used.^19^ This double sandwich electrochemiluminescence immunoassay uses a recombinant protein of the receptor binding domain on the spike protein as an antigen for the determination of antibodies against SARS-CoV-2. Sample electrochemiluminescence signals are compared with internal calibration curves and quantitative values are reported as units (U) per mL. In-house validation experiments have been described previously.^13^ An additional dilution step was added for samples with antibody concentrations greater than the analytical range of the assay following the third vaccine dose. Anti-SARS-CoV-2 spike (S1 receptor binding domain [RBD]) antibody concentrations were measured at 53–92 days after the second vaccine dose and 28–49 days after the third vaccine dose.

At entry to the VIP study (at 53–92 days after the second vaccine dose) and at 28–49 days after the third vaccine dose, all participants were tested for possible previous SARS-CoV-2 infection using the Elecsys anti-SARS-CoV-2 N immunoassay (Roche, Rotkreuz, Switzerland). A concentration of greater than or equal to 0·12 U/mL was defined as a threshold below which participants were deemed to have no evidence of previous infection. Participants who reported a history of a previous positive PCR test confirming SARS-CoV-2 infection at any time were recorded as being previously SARS-CoV-2 infected.

At 28–49 days after the third vaccine dose, whole blood was collected in lithium heparin tubes and peripheral blood mononuclear cells were isolated by density-gradient centrifugation using Lymphoprep (Stem Cell Technologies, Vancouver, BC, Canada) layered onto SepMate (Stem Cell Technologies) tubes. Isolation of peripheral blood mononuclear cells was done within 12 h of venepuncture. Purified peripheral blood mononuclear cells were cryopreserved in fetal bovine serum supplemented with 10% dimethyl sulphoxide and stored in liquid nitrogen pending batch analysis.

T cell analysis was done at the Department of Infectious Disease, Faculty of Medicine, Imperial College London. T cells were measured 28–49 days after the third vaccine dose. IFN-γ T-cell ELISpot assays were done using precoated plates (Mabtech 3420-2APT, MabTech, Nacka Strand, Sweden) and using the protocol described previously.^6^, ^20^, ^21^ 200 000 cells were seeded per well and cells were stimulated with a peptide pool, containing 18 peptides derived from SARS-CoV-2 spike protein^22^ at a concentration of 10 μg/mL per peptide. The peptide pool uses a mapped epitope pool of 12–20mer peptides, mapped as eliciting high-prevalence CD4 responses covering diverse HLA-II haplotypes.^20^, ^21^ Use of this spike mapped epitope pool in otherwise healthy SARS-CoV-2 seropositive individuals elicits a T-cell response in 83% of individuals at 16–18 weeks after natural SARS-CoV-2 infection and 91% of healthy individuals 2–3 weeks after two-dose vaccination, with seronegative individuals showing a level of response indistinguishable from prepandemic controls.^20^, ^21^ Plates were cultured for 18–20 h before development and data were collected using an AID classic ELISpot plate reader (Autoimmun Diagnostika, Strassberg, Germany). Results are expressed as differences in spot-forming cells per 10^6^ peripheral blood mononuclear cells between peptide stimulation and a media-only control. A response falling below 2 SDs above the media-only control wells was deemed to be a null response. Data were excluded if the response to the positive control anti-CD3 stimulation was less than 200 spot-forming cells per 10^6^ peripheral blood mononuclear cells.

Variables self-reported and recorded by participants at the time of study enrolment were demographics (age, sex, ethnicity, comorbidities, height, bodyweight, smoking status, and postcode), IBD disease activity (defined by patient-reported outcomes [PRO2] at study enrolment),^23^, ^24^ SARS-CoV-2 symptoms aligned to the COVID-19 symptoms study (symptoms, previous testing, and hospital admissions for COVID-19) and vaccine uptake (type and date of primary vaccination). Data were entered electronically into a purpose-designed REDCap database hosted at the Royal Devon and Exeter NHS Foundation Trust.^25^ An additional questionnaire was administered after the third vaccine dose to capture third dose vaccination type, positive COVID-19 tests between the second and third dose, and changes in IBD treatment. Participants without access to the internet or electronic device completed their questionnaires on paper case record forms that were subsequently entered by local research teams.

### Outcomes

The primary outcome was anti-SARS-CoV-2 S1 RBD antibody concentration in each study group (the six immunosuppressive therapy groups plus the healthy control group) 28–49 days after the third vaccine dose, adjusted by age, homologous versus heterologous vaccine schedule, and previous SARS-CoV-2 infection.

Secondary outcomes were the relative increment in anti-SARS-CoV-2 S1 RBD antibody concentrations following the third vaccine dose in each study group (the six immunosuppressive therapy groups plus the healthy control group), and spike-peptide specific T-cell responses in each group following the third vaccine dose.

All outcomes were centrally assessed. Outcomes relating to anti-SARS-CoV-2 S1 RBD antibody concentrations were assessed in all participants. T cell responses were assessed in all participants with available data.

### Statistical analysis

Sample size calculations for the VIP study have been reported previously.^5^ Full details can be found in the statistical analysis plan. All tests were two-tailed and values of p less than 0·05 were considered significant. We included patients with missing clinical data in analyses for which they had data and have specified the denominator for each variable. No imputation of missing data was done. Anti-SARS-CoV-2 S1 RBD antibody concentrations are reported as geometric means and SD (Geometric SD[*x*]=*e*^SD[logx]^). Other continuous data are reported as median and IQR, and discrete data as numbers and percentages, unless otherwise stated.

For the primary outcome analysis, linear regression models of log-transformed anti-SARS-CoV-2 S1 RBD antibody concentration, adjusted for age, vaccine schedule, and previous SARS-CoV-2 infection (adjustments made owing to the substantial effect of these variables on humoral responses to SARS-CoV-2 vaccination), were used to identify IBD treatment regimens associated with the concentration of anti-SARS-CoV-2 S1 RBD antibody. To test our primary outcome, we used multivariable linear regression models to assess the association between immunosuppressive therapies in IBD and COVID-19 vaccine-induced antibody responses, adjusted for confounders. On the basis of data from CLARITY-IBD, a priori, we included IBD medication, vaccine type (mRNA or adenovirus), age, IBD subtype, ethnicity, and smoking status.^13^ Age was treated as a continuous variable in the analysis (after checking the linearity of age as a variable using simple linear regression and Runs test) and its coefficient is expressed per decade. Results are presented after exponentiation, so that the exponentiated coefficients of the model correspond to the geometric mean ratio (GMR) estimates per one unit increase associated with each binary covariate. Our analysis for the multivariable linear regression model assumed that the anti-SARS-CoV-2 S1 RBD antibody data would be log normally distributed. Model diagnostics were performed to test this assumption. We subsequently did a sensitivity analysis using a one-parameter Box-Cox transformation^26^ with λ=0·2 (based on optimising the log-likelihood of the model) to ensure that data skew did not substantially affect our results. In addition, to account for the within-patient multiple measurements of anti-SARS-CoV-2 S1 RBD antibody concentration (at visit 1 and visit 2), a linear mixed-effects model was also used, including data from visit 1 and visit 2. The linear mixed-effects model was fitted using the lmer package^27^ with log(antibody concentration) as the outcome variable, the participant as a random variable for the intercept, and fixed variables as specified in the results table. The error distribution was assumed to be normal, and this assumption was checked by visual inspection of a Q–Q plot of the residuals. Wilcoxon matched-pairs signed-rank tests were used for comparison of anti-SARS-CoV-2 S1 RBD antibody concentrations after the second and third vaccine dose stratified by treatment group.

Kruskal-Wallis tests, with Dunn's correction for multiple testing, were used to compare the magnitude of T-cell responses (spot forming cells per 10^6^ peripheral blood mononuclear cells) stratified by immunosuppressive therapy and previous SARS-CoV-2 infection. Spearman's rank correlation coefficient was calculated to determine the correlation between antibody and T-cell responses. Statistical analyses were undertaken in R version 4.0.4. Figures were created in R version 4.0.4 and Graphpad Prism version 9.0.0. This study is registered with ISRCTN, ISRCTN13495664.

### Role of the funding source

The funder of the study had no role in study design, data collection, data analysis, data interpretation, or writing of the report.

## Results

Between Oct 18, 2021, and March 29, 2022, 352 participants were included in the study following a third dose of SARS-CoV-2 vaccine (participants being treated with thiopurine n=65, infliximab n=46, thiopurine plus infliximab combination therapy n=49, ustekinumab n=44, vedolizumab n=50, tofacitinib n=26, and healthy controls n=72; table). 125 (36%) participants had evidence of previous SARS-CoV-2 infection. Missing clinical data affected four (1%) of 352 patients included in the analysis of the primary outcome (all four in the vedolizumab group); therefore, these patients were excluded from the multivariable model.

**Table:** Characteristics of participants attending the second study visit

		**Thiopurine (n=65)**	**Infliximab (n=46)**	**Thiopurine plus infliximab (n=49)**	**Ustekinumab (n=44)**	**Vedolizumab (n=50)**	**Tofacitinib (n=26)**	**Healthy control (n=72)**	**p value**
Previous SARS-CoV-2 infection		..	..	..	..	..	..	..	0·59
	Neither swab nor serology	43 (66%)	30 (65%)	29 (59%)	31 (70%)	35 (70%)	15 (58%)	44 (61%)	..
	Swab	3 (5%)	4 (9%)	1 (2%)	1 (2%)	1 (2%)	0	0	..
	Serology	19 (15%)	9 (20%)	15 (31%)	8 (18%)	10 (20%)	7 (27%)	18 (25%)	..
	Swab and serology	9 (14%)	3 (7%)	4 (8%)	4 (9%)	4 (8%)	4 (15%)	10 (14%)	..
Age, years		44·1 (34·6–54·5)	47·5 (36·1–56·4)	39·2 (31·1–52·1)	43·6 (33·1–56·4)	44·6 (37·0–59·2)	48·0 (37·9–54·8)	36·5 (29·0–50·6)	0·029
Gender		..	..	..	..	..	..	..	0·0085
	Female	36 (55%)	22 (48%)	24 (49%)	23 (52%)	15 (33%)*	8 (31%)	47 (65%)	..
	Male	29 (45%)	24 (52%)	25 (51%)	21 (48%)	31 (67%)*	18 (69%)	25 (35%)	..
	Other	0	0	0	0	0*	0	0	..
	Prefer not to say	0	0	0	0	0*	0	0	..
Non-White ethnicity		12 (18%)	8 (17%)	19 (20%)	5 (11%)	11 (24%)*	4 (15%)	12 (17%)	0·84
Ethnicity		..	..	..	..	..	..	..	0·91
	White	53 (82%)	38 (83%)	39 (80%)	39 (89%)	35 (76%)*	22 (85%)	60 (83%)	..
	Asian	7 (11%)	4 (9%)	7 (14%)	4 (9%)	7 (15%)*	2 (8%)	8 (11%)	..
	Mixed	0	2 (4%)	2 (4%)	1 (2%)	2 (4%)*	1 (3%)	3 (4%)	..
	Black	2 (3%)	0	1 (2%)	0	1 (2%)*	0	0	..
	Other	3 (5%)	2 (4%)	0	0	1 (2%)*	1 (4%)	1 (1%)	..
Diagnosis		..	..	..	..	..	..	..	0·0005
	Crohn's disease	28 (43%)	31 (67%)	30 (61%)	43 (98%)	22 (44%)	2 (8%)	NA	..
	Ulcerative colitis	36 (55%)	13 (28%)	16 (33%)	1 (2%)	27 (54%)	24 (92%)	NA	..
	Unclassified inflammatory bowel disease	1 (2%)	2 (4%)	3 (6%)	0	1 (2%)	0	NA	..
BMI, kg/m^2^		24·2 (21·8–27·4)	25·2 (23·3–28·5)	25·1 (22·4–26·9)	25·7 (22·8–29·8)	25·0 (23·1–28·4)	25·3 (23·0–28·6)	23·4 (21·7–25·7)	0·067
Heart disease		1 (2%)	1 (2%)	0	0	3 (7%)*	0	0	0·089
Diabetes		4 (6%)	3 (7%)	0	3 (7%)	3 (7%)*	0	1 (1%)	0·22
Lung disease		7 (11%)	7 (15%)	7 (14%)	4 (9%)	3 (7%)*	3 (12%)	6 (8%)†	0·81
Kidney disease		1 (2%)	2 (4%)	0	1 (2%)	1 (2%)*	0	0	0·44
Cancer		1 (2%)	1 (2%)	0	0	1 (2%)*	0	0	0·65
Smoker		..	..	..	..	..	..	..	0·25
	Yes	1 (2%)	2 (4%)	2 (4%)	3 (7%)	5 (11%)*	2 (8%)	2 (3%)	..
	Not currently	23 (35%)	13 (28%)	16 (33%)	15 (34%)	15 (33%)*	13 (50%)	17 (24%)	..
	Never	41 (63%)	31 (67%)	31 (63%)	26 (59%)	26 (57%)*	11 (42%)	53 (74%)	..
Vaccine doses one and two		..	..	..	..	..	..	..	0·023
	BNT162b2 (Pfizer) vaccine	25 (38%)	26 (57%)	16 (33%)	15 (34%)	17 (37%)*	7 (27%)	35 (49%)	..
	ChAdOx1-S (AstraZeneca) vaccine	40 (62%)	20 (43%)	33 (67%)	29 (66%)	29 (63%)*	18 (69%)	33 (46%)	..
	mRNA-1273 (Moderna) vaccine	0	0	0	0	0*	1 (4%)	4 (6%)	..
Prednisolone		2 (3%)‡	4 (9%)	3 (6%)	2 (5%)	4 (9%)*	4 (15%)	NA	0·41
Immunosuppressive therapy stopped or switched at time of third dose		1 (2%)	3 (7%)	5 (10%)	2 (5%)	2 (4%)	1 (4%)	NA	0·44
Active disease (PRO2)		6 (9%)	1 (2%)	2 (4%)§	3 (8%)¶	8 (19%)‖	2 (8%)**	NA	0·11
Days since third dose of vaccine		39·0 (33·0–44·0)	40·0 (35·0–46·0)	39·0 (36·0–44·5)	39·0 (33·5–44·5)	40·0 (34·7–43·8)	35·5 (32·0–40·5)	39·0 (34·0–44·5)	0·49

Data are median (IQR) or n (%). p values were obtained using Fisher's exact tests for categorical variables and Kruskal-Wallis tests for continuous variables. NA=not applicable.

* N=46.

† N=71.

‡ N=64.

§ N=47.

¶ N=40.

‖ N=43.

** N=25.

**Figure 1:**
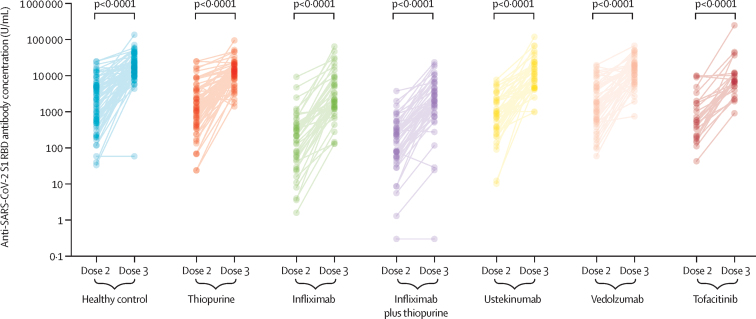
Ladder plots showing anti-SARS-CoV-2 S1 RBD antibody binding after two doses and three doses of COVID-19 vaccine, stratified by study treatment group Statistical analysis was performed with Wilcoxon signed-rank test. RBD=receptor binding domain.

We first compared anti-SARS-CoV-2 S1 RBD antibody concentrations in individuals stratified by immunosuppressive therapy after the second and third vaccine doses (figure 1). Geometric mean anti-SARS-CoV-2 S1 RBD antibody concentrations were significantly higher in the healthy control group and all treatment groups following a third dose of vaccine than following the second dose of vaccine (all p<0·0001).

**Figure 2:**
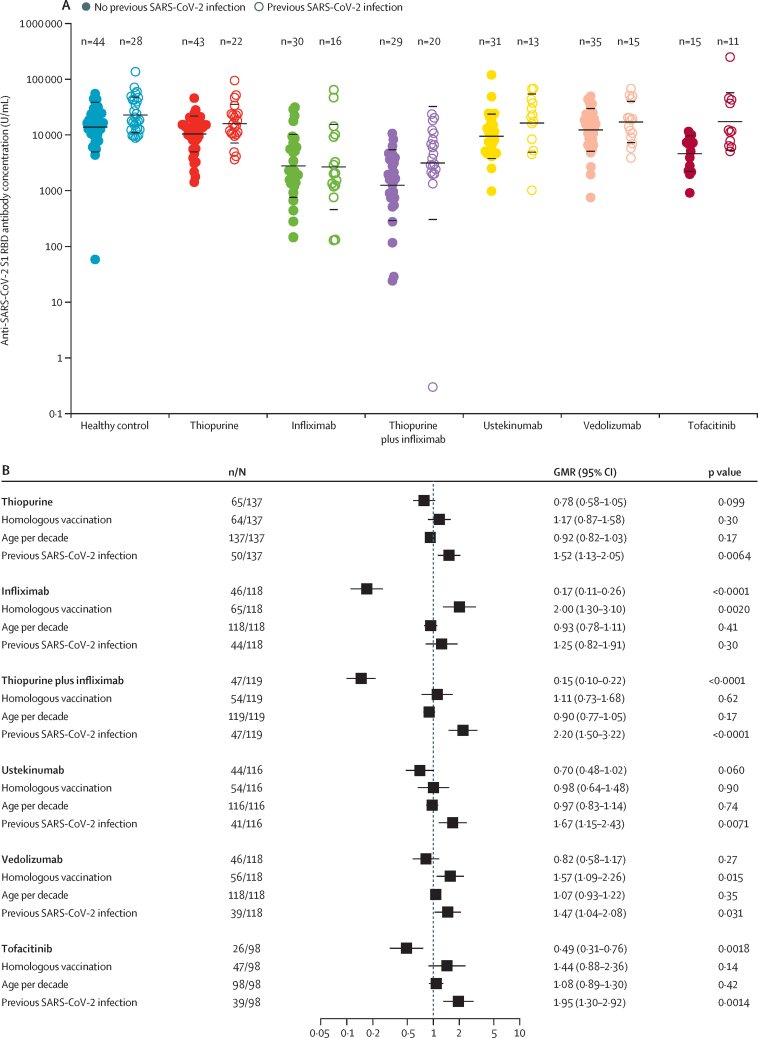
Anti-SARS-CoV-2 spike protein antibody concentrations in the healthy control group and patients with inflammatory bowel disease who are triple vaccinated against COVID-19 (A) SARS-CoV-2 spike S1 RBD antibody binding 28–49 days after the third dose of vaccine, stratified by study treatment group and previous infection. The wider bar represents the geometric mean; the narrower bars are one geometric SD either side of the geometric mean. (B) Multivariable models showing coefficients of linear regression models of log(anti-SARS-CoV-2 S1 RBD antibody concentration) stratified by study treatment group. GMR=geometric mean ratio. RBD=receptor binding domain.

Geometric mean anti-SARS-CoV-2 S1 RBD antibody concentrations were lower in patients treated with infliximab (2736·8 U/mL [geometric SD 4·3]; p<0·0001), infliximab plus thiopurine (1818·3 U/mL [6·7]; p<0·0001) and tofacitinib (8071·5 U/mL [3·1]; p=0·0018) compared with the healthy control group (16 774·2 U/mL [2·6]; figure 2). No significant differences in anti-SARS-CoV-2 S1 RBD antibody binding were found between the healthy control group and patients treated with thiopurine (12 019·7 U/mL [2·2]; p=0·099), patients treated with vedolizumab (13 564·9 U/mL [2·4]; p=0·27), or patients treated with ustekinumab (11 089·3 U/mL [2·8]; p=0·060). One patient treated with infliximab plus thiopurine therapy did not mount a detectable antibody response. Anti-SARS-CoV-2 S1 RBD antibody binding for each vaccine schedule type (three doses mRNA [homologous] and two doses adenovirus vector and one dose mRNA [heterologous]) stratified by study group are shown in the appendix (pp 3–4).

**Figure 3:**
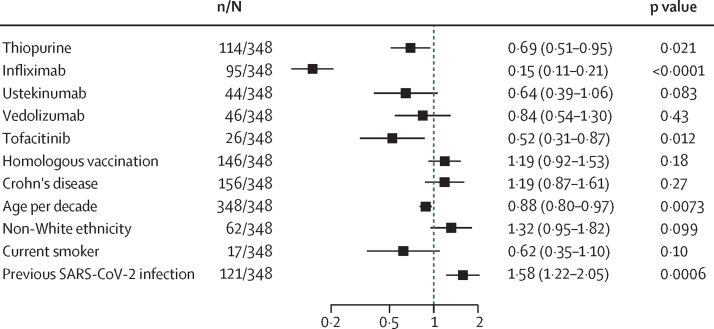
Multivariable model showing exponentiated coefficients of linear regression models of log(anti-SARS-CoV-2 spike S1 RBD antibody binding) The values shown represent GMRs of anti-SARS-CoV-2 S1 RBD binding associated with each variable. Age was treated as a continuous variable in the analysis and its coefficient is expressed per decade. GMR=geometric mean ratio.

In multivariable modelling, lower anti-SARS-CoV-2 S1 RBD antibody concentrations were independently associated with infliximab and tofacitinib, but not with vedolizumab (figure 3). The model also suggests that thiopurine might be associated with modest reductions in anti-SARS-CoV-2 S1 RBD antibody concentration**.** The association between ustekinumab and anti-SARS-CoV-2 S1 RBD antibody concentrations was also suggestive of a modest reduction, but these results were not statistically significant. Previous SARS-CoV-2 infection was independently associated with higher anti-SARS-CoV-2 S1 RBD antibody concentrations, and older age was independently associated with lower anti-SARS-CoV-2 S1 RBD antibody concentrations. Homologous vaccination schedule, IBD subtype, ethnicity, and smoking status were not associated with anti-SARS-CoV-2 S1 RBD antibody binding. A linear mixed effects model, additionally adjusting for within-patient multiple measurements showed no significant effect on the reported associations (appendix p 1). After running diagnostics to test statistical assumptions underlying the multivariable model (appendix p 6), a one-parameter Box-Cox transformation (appendix p 7) with λ=0·20 (based on optimising the log-likelihood of the model), showed no significant effect on the treatment variables in the multivariable linear regression model (appendix p 8).

**Figure 4:**
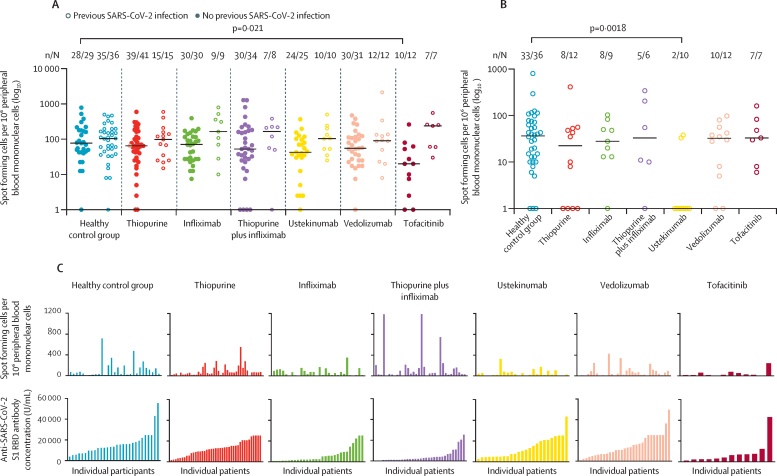
T-cell immunity against SARS-CoV-2 spike and nucleocapsid in the healthy control group and patients with IBD who are triple vaccinated against COVID-19 (A) T-cell responses against SARS-CoV-2 spike mapped epitope pool. (B) T-cell responses against SARS-CoV-2 nucleocapsid mapped epitope pool. For panels A and B, statistical significance was determined using a Kruskal-Wallis multiple comparison test with Dunn's correction; previously infected donors were assayed for nucleocapsid T-cell responses; the number of study participants in each group with a positive T-cell response to the peptide pools is shown; and n/N=number of T-cell responders/number of individuals tested. Midlines on both A and B are the geometric means. (C) Individual donor T-cell responses to the spike mapped epitope pool and matched data for serum S1 RBD binding antibodies, plotted by ascending antibody binding titer for SARS-CoV-2 infection-naive healthy control group (n=29) and SARS-CoV-2 infection-naive patients with inflammatory bowel disease taking thiopurine (n=41), infliximab (n=30), thiopurine plus infliximab (n=34), ustekinumab (n=25), vedolizumab (n=31), or tofacitinib (n=12). RBD=receptor binding domain.

In 53 (15%) patients (thiopurine n=9, infliximab n=7, thiopurine plus infliximab n=7, ustekinumab n=9, vedolizumab n=7, tofacitinib n=7, and healthy controls n=7), T-cell responses could not be reported, either due to insufficient blood draw, insufficient cell number harvest during peripheral blood mononuclear cell extraction, or technical failure of the assay. In participants without evidence of previous SARS-CoV-2 infection, the magnitude of anti-SARS-CoV-2 spike T-cell responses was lower in patients treated with tofacitinib than in the healthy control group (p=0·021; figure 4A). No significant differences in the magnitude of anti-SARS-CoV-2 spike T-cell responses were observed in infection-naive recipients of thiopurine (p>0·99), infliximab (p>0·99), thiopurine plus infliximab (p>0·99), ustekinumab (p=0·42), or vedolizumab (p>0·99), compared with the healthy control group. In individuals with laboratory confirmed evidence of previous SARS-CoV-2 infection, there were no differences observed in the magnitude of anti-SARS-CoV-2 spike T-cell responses between the groups (figure 4A; appendix p 2). In individuals with evidence of previous infection, T-cell responses against the SARS-CoV-2 nucleocapsid mapped epitope pool were significantly lower in patients treated with ustekinumab than in the healthy control group (p=0·0018; figure 4B). There were no significant differences observed in the magnitude of T-cell responses against SARS-CoV-2 nucleocapsid mapped epitope pool between the other treatment groups and the healthy control group (figure 4B; appendix p 2). Ordering anti-SARS-CoV-2 spike T-cell responses by the cumulative magnitude of anti-SARS-CoV-2 S1 RBD binding following three doses of COVID-19 vaccine showed discordant T-cell and antibody responses in all treatment groups (figure 4C).

## Discussion

This study provides new information on the effect of different commonly used immunosuppressive drugs on T-cell and antibody responses after three doses of COVID-19 vaccine. The first key finding is that patients with IBD on each of the six treatment regimens studied gain a significant boost in antibody binding levels from a third vaccine dose, supporting the decision taken in many countries to roll out third primary doses of vaccine to these groups. However, patients treated with infliximab or tofacitinib had reduced anti-SARS-CoV-2 S1 RBD antibody binding after three doses of vaccine compared with a healthy control group. Patients with IBD on thiopurine monotherapy, ustekinumab, or vedolizumab showed no significant reduction in antibody binding compared with control participants. These findings mirror differences seen in the previously reported VIP study following two doses of vaccine.^5^

The size of reduction in antibody binding was greatest in patients treated with infliximab, with an 84% reduction in antibody binding when compared with participants in the control group. These findings are compatible with observations after the third vaccine dose in the CLARITY-IBD,^28^ PREVENT-COVID,^29^ and HERCULES studies,^30^ but contrast with a recent Canadian study in which anti-TNF therapy was not associated with a significant reduction in anti-SARS-CoV-2 spike antibody titre following three doses of vaccine.^11^ Notably, the Canadian study enrolled 16 non-immunosuppressed patients with a diagnosis of IBD rather than healthy controls as a reference group.^11^ Despite the relative reduction in antibody binding seen in patients treated with anti-TNF, our results are better than those seen in some other immunosuppressed groups, such as recipients of solid organ transplants, a substantial minority of whom do not mount any detectable response to a third vaccine dose.^31^ Reassuringly for recipients of infliximab, our results also showed that T-cell responses following three doses of vaccine were not reduced relative to the healthy control group. These data are in line with observations from CLARITY-IBD, in which T-cell responses were not significantly different between patients treated with infliximab and patients treated with vedolizumab following two doses of vaccine,^6^ but we have not recapitulated the findings of the CORALE study, which showed augmentation of T-cell response in recipients of anti-TNF.^32^ In the current study, we observed that patients treated with thiopurine, infliximab, thiopurine plus infliximab, ustekinumab, or vedolizumab did not differ significantly from healthy controls in terms of T-cell response. However, tofacitinib treatment was associated with reduced T-cell immunity against spike protein, suggesting that this treatment impairs humoral and cell-mediated response to COVID-19 vaccination, which might mark out patients on this treatment as particularly susceptible during future waves of SARS-CoV-2 infection. In the omicron (B.1.1.529) era, with postvaccination breakthrough infection and re-infection increasingly common in immunosuppressed and non-immunosuppressed groups, translating studies of vaccine immunogenicity into practice will continue to challenge clinicians and policy makers. Studies are urgently needed to assess the relative immunogenicity of vaccines against emerging variants of concern in immunosuppressed patients with IBD, and to determine how immunogenicity corresponds to risk of severe disease and death.

Although our study has strengths, including a large, well balanced cohort and both humoral and cell-mediated readouts of vaccine response, we acknowledge limitations. First, the number of participants in the tofacitinib group is small, and we should interpret findings in this group with caution. Modest reductions in SARS-CoV-2 antibody binding observed in the thiopurine and ustekinumabs group were not statistically significant. Based on these results, although we cannot be certain that thiopurines and ustekinumab are not associated with a reduction in serological response, any differences from the healthy population are unlikely to be clinically important. In multivariable modelling, we have accounted for important confounding factors associated with humoral responses to vaccination in other studies (including age, vaccine type, IBD subtype, smoking status, ethnicity, previous infection, and heterologous vaccination schedules). However, confounders were not selected using a causal directed acyclic graph, and we cannot exclude the possibility that our results are affected by measurement bias or residual confounding due to measurement error in the outcome variable and other measured or unmeasured confounders. IBD disease activity was assessed clinically using PRO2 and did not differ significantly between treatment groups, but we do not have information on biochemical or endoscopic activity. Previous SARS-CoV-2 infection was treated as a binary variable, but it is possible that infection with SARS-CoV-2 variants of concern during different waves of the pandemic differentially shape immunity.^33^

In conclusion, we have shown that three doses of COVID-19 vaccine provided a significant boost in vaccine-induced antibody binding in patients taking various immunosuppressive treatments commonly used in IBD, but that patients treated with infliximab or tofacitinib showed reduced antibody binding relative to a healthy control group. Patients on tofacitinib additionally showed reduced vaccine-induced T-cell immunity against ancestral spike, raising the question of whether this group is particularly susceptible to infection by SARS-CoV-2. Notably, vaccine-induced immunity after three doses of vaccine was greater in participants who had previously been infected with SARS-CoV-2, consistent with the notion that further antigen exposure could rescue suboptimal responses.^21^ It is possible that additional doses of vaccine recover immunity in those patients taking immunosuppressive treatments linked to suboptimal vaccine immunogenicity, such as infliximab or tofacitinib.

## Data sharing

The study protocol including the statistical analysis plan is available at www.vipstudy.uk. All individual participant de-identified data that underlie the results reported in this article will be available immediately after publication. The de-identified data will be made available indefinitely to anyone who wishes to access the data at https://doi.org/10.5281/zenodo.7054354.

## Declaration of interests

JLA reports sponsorship from Vifor Pharma for accommodation and travel to BSG 2019, outside the submitted work. NAK reports grants from AbbVie, Biogen, Celgene, Celtrion, Galapagos, MSD, Napp, Pfizer, Pharmacosmos, Roche, and Takeda; consulting fees from Amgen, Bristol Myers Squibb, Falk, Janssen, Mylan, Pharmacosmos, Galapagos, Takeda, and Tillotts; personal fees from Allergan, Celltrion, Falk, Ferring, Janssen, Pharmacosmos, Takeda, Tilllotts, and Galapagos; and support for attending meetings from AbbVie, Falk, and Janssen, outside the submitted work. AS has received travel expense support from Janssen. SS reports grants from Takeda, AbbVie, Tillots Pharma, Janssen, Pfizer, and Biogen; and personal fees from Takeda, AbbVie, Janssen, Pharmacocosmos, Biogen, Pfizer, Tillots Pharma, and Falk Pharma, outside the submitted work. ALH reports payment or honoraria for lectures, presentations, speakers bureaus, manuscript writing, or educational events from AbbVie, AstraZeneca, Atlantic, Bristol Myers Squibb, Celltrion, Falk, Galapogos, Janssen, MSD, Napp Pharmaceuticals, Pfizer, Pharmacosmos, Shire, and Takeda; global steering committee for Genentech; support for attending meetings from AbbVie, Takeda, and Janssen; and participation on a data safety monitoring board or advisory board for AbbVie, AstraZeneca, Atlantic, Bristol Myers Squibb, Galapogos, Janssen, Pfizer, and Takeda. PMI reports grants and personal fees from Celltrion, Takeda, Pfizer, Galapagos; grants from MSD; and personal fees from Gilead, AbbVie, Janssen, Bristol Myers Squibb, Lilly, and Arena, outside the submitted work. MP receives unrestricted educational grants from Pfizer for genetic analyses to support the IBD BioResource, and speaker fees from Janssen. GRJ has received grants from Wellcome Trust and ECCO; speaker fees from Takeda, Ferring, and Janssen; and support for attending meetings or travel from Ferring. KK reports payment or honoraria for lectures, presentations, speakers bureaus, manuscript writing, or educational events from Janssen and Ferring; support for attending meetings or travel from Janssen and Takeda; and participation on a data safety monitoring board or advisory board for Janssen and Predict Immune. SB reports funding from Ferring and Dr Falk for accommodation, travel, and meeting fees. KVP reports payment or honoraria for lectures, presentations, speakers bureaus, manuscript writing, or educational events from AbbVie, Dr Falk, Janssen, PreddictImmune, and Takeda; support for attending meetings or travel from AbbVie, Ferring, Janssen, and Tillots; and participation on a data safety monitoring board or advisory board for AbbVie, Galapagos, and Janssen. AJK reports consulting fees from Janssen; payment or honoraria for lectures, presentations, speakers bureaus, manuscript writing, or educational events from Pfizer and Takeda; support for attending meetings or travel from Janssen, Tillots, and Norgine; and participation on a data safety monitoring board or advisory board for AbbVie. LCH reports support for attending meetings or travel from AbbVie. CWL reports a Future Leaders Fellow award from UKRI; personal consulting fees from Galapagos, AbbVie, Takeda, Pfizer, Janssen, and Iterative Scopes; institutional consulting fees from Trellus Health; personal fees from Galapagos, AbbVie, Takeda, Pfizer, Janssen, GSK, Gilead, Fresnius Kabi, Ferring, and Dr Falk; and support for attending meetings from Galapagos, AbbVie, Takeda, Pfizer, Janssen, GSK, Gilead, Fresnius Kabi, Ferring, and Dr Falk. RJB and DMA are members of the Global T cell Expert Consortium and have consulted for Oxford Immunotec outside the submitted work. JRG reports grants from F Hoffmann-La Roche AG; grants from Biogen, Celltrion Healthcare, and Galapagos NV; and non-financial support from Immundiagnostik, during the conduct of the study. TA reports grant funding from Pfizer to his institution to deliver this study; grants from Celltrion, Roche, Takeda, Biogen, and Galapagos; and honoraria for lectures from Takeda and Roche, outside the submitted work. Financial support for the VIP study was provided as a research grant by Pfizer and NP is the principal investigator on this grant. NP has received research grants from Bristol Myers Squibb outside the submitted work; reports personal fees from Takeda, Janssen, Pfizer, Bristol Myers Squibb, AbbVie, Roche, Lilly, Allergan, and Celgene, outside the submitted work; and has served as a speaker or advisory board member for AbbVie, Allergan, Bristol Myers Squibb, Celgene, Falk, Ferring, Janssen, Pfizer, Tillotts, Takeda, and Vifor Pharma. All other authors declare no competing interests.
