# Supplementary material for: COVID-19 vaccine-induced antibody and T-cell responses in immunosuppressed patients with inflammatory bowel disease after the third vaccine dose (VIP): a multicentre, prospective, case-control study
Source: Lancet Gastroenterol Hepatol. 2022 Sep 9;7(11):1005–15. doi: 10.1016/S2468-1253(22)00274-6 (PMC9458592; doi:10.1016/S2468-1253(22)00274-6)
Supplement: Supplementary appendix [file mmc1.pdf]

THE LANCET
Gastroenterology & Hepatology

Supplementary appendix

This appendix formed part of the original submission and has been peer reviewed. We post it as supplied by the authors.

Supplement to: Alexander JL, Liu Z, Sandova DM, et al. COVID-19 vaccine-induced antibody and T-cell responses in immunosuppressed patients with inflammatory bowel disease after the third vaccine dose (VIP): a multicentre, prospective, case-control study. *Lancet Gastroenterol Hepatol* 2022; published online Sept 8. https://doi.org/10.1016/S2468-1253(22)00274-6.

COVID-19 vaccine-induced antibody and T cell responses in immunosuppressed patients with inflammatory bowel disease after the third vaccine dose (VIP): a multicentre, prospective, case-control study

Supplementary Material

Supplementary table 1: Linear mixed effects model including visit 1 (post second vaccine dose) and visit 2 (post third vaccine dose) anti-S1 RBD antibody concentrations. Study visit was analysed as a fixed effect.

Variable	Estimate	95% CIs	P-value
Thiopurine	0.85	0.64 - 1.14	0.29
Infliximab	0.13	0.09 - 0.18	<0.0001
Ustekinumab	0.72	0.45 – 1.15	0.17
Vedolizumab	1.04	0.69 - 1.56	0.86
Tofacitinib	0.57	0.35 – 0.92	0.021
Visit: visit 2	16.75	13.94 – 20.13	<0.0001
mRNA vaccine effect on visit 1	3.30	2.50 – 4.34	<0.0001
Baseline mRNA vaccine effect on visit 2 (i.e. homologous vaccination)	1.19	0.90 – 1.56	0.22
Crohn's disease	1.08	0.81 – 1.44	0.60
Age (per decade)	0.84	0.77 – 0.91	<0.0001
Non-white ethnicity	1.05	0.78 – 1.43	0.74
Current smoker	0.67	0.39 – 1.15	0.15
Prior infection (assessed separately for each visit)	2.27	1.80 – 2.87	<0.0001

Supplementary table 2: T cell immunity against SARS-CoV-2 spike in triple COVID-19 vaccinated IBD patients and healthy controls with evidence of prior infection. P values calculated using Kruskal Wallis multiple comparison test with Dunn’s correction.

**Comparison**	**Mean rank difference**	**P-value**
Healthy control vs. Thiopurine	1.11	>0.99
Healthy control vs. Infliximab	-13.01	>0.99
Healthy control vs. Thiopurine + Infliximab	-6.21	>0.99
Healthy control vs. Ustekinumab	-2.16	>0.99
Healthy control vs. Vedolizumab	2.04	>0.99
Healthy control vs. Tofacitinib	-5.58	>0.99

Supplementary table 3: T cell immunity against SARS-CoV-2 nucleocapsid in triple COVID-19 vaccinated IBD patients and healthy controls with evidence of prior infection. P values calculated using Kruskal Wallis multiple comparison test with Dunn’s correction.

**Comparison**	**Mean rank difference**	**P-value**
Healthy control vs. Thiopurine	10.56	>0.99
Healthy control vs. Infliximab	1.65	>0.99
Healthy control vs. Thiopurine + Infliximab	-0.99	>0.99
Healthy control vs. Ustekinumab	33.98	0.0018
Healthy control vs. Vedolizumab	6.43	>0.99
Healthy control vs. Tofacitinib	-0.99	>0.99

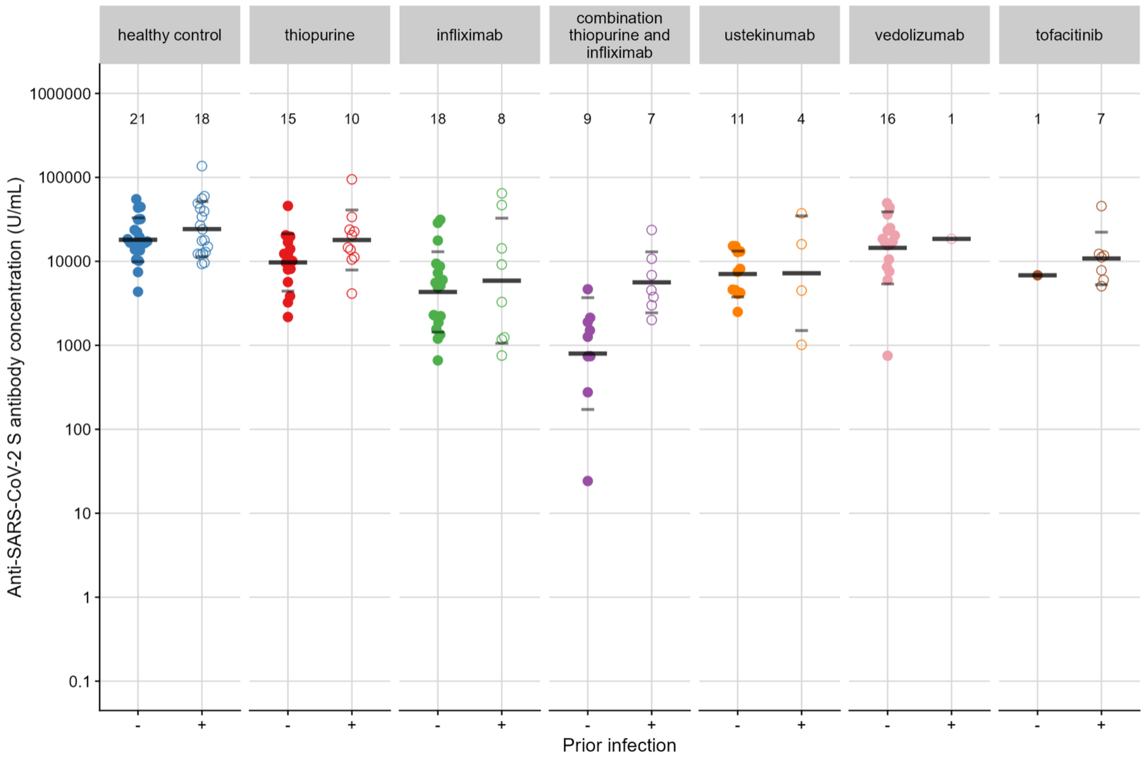

Supplementary figure 1: Anti-SARS-CoV-2 spike antibody concentration in participants receiving a homologous vaccine schedule (three doses of mRNA vaccine) stratified by study group and previous infection status. The wider bar represents the geometric mean, while the narrower bars are one geometric SD either side of the geometric mean.

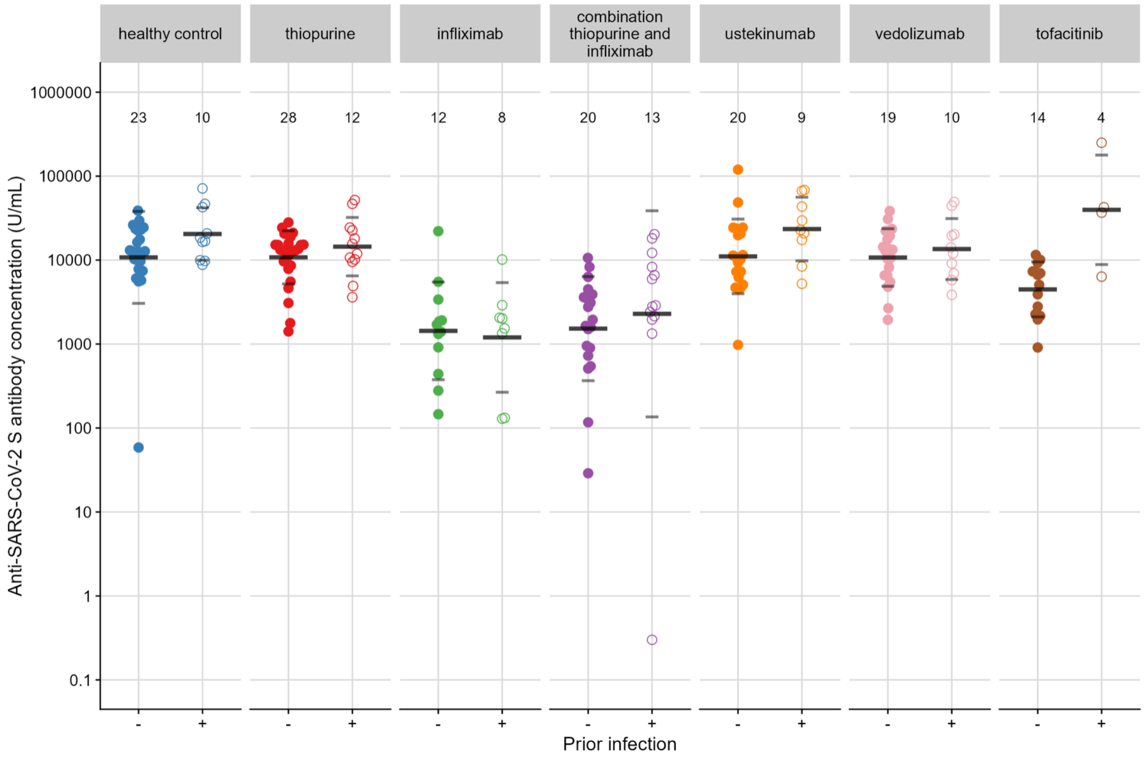

Supplementary figure 2: Anti-SARS-CoV-2 spike antibody concentration in participants receiving a heterologous vaccine schedule (two doses of adenovirus vector vaccine and one dose of mRNA vaccine) stratified by study group and previous infection status. The wider bar represents the geometric mean, while the narrower bars are one geometric SD either side of the geometric mean.

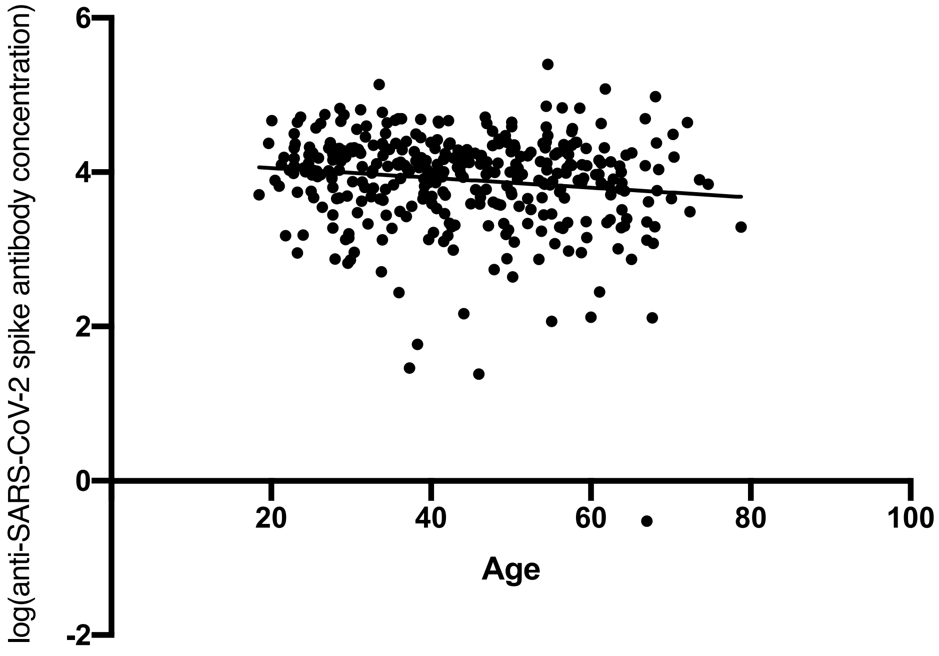

Supplementary figure 3: Simple linear regression model of age versus log[anti-SARS-CoV-2 spike antibody concentration] (R^2=0.02; p=0.0091). Runs test for deviation from linearity (p=0.67) indicated non-deviation from linearity.

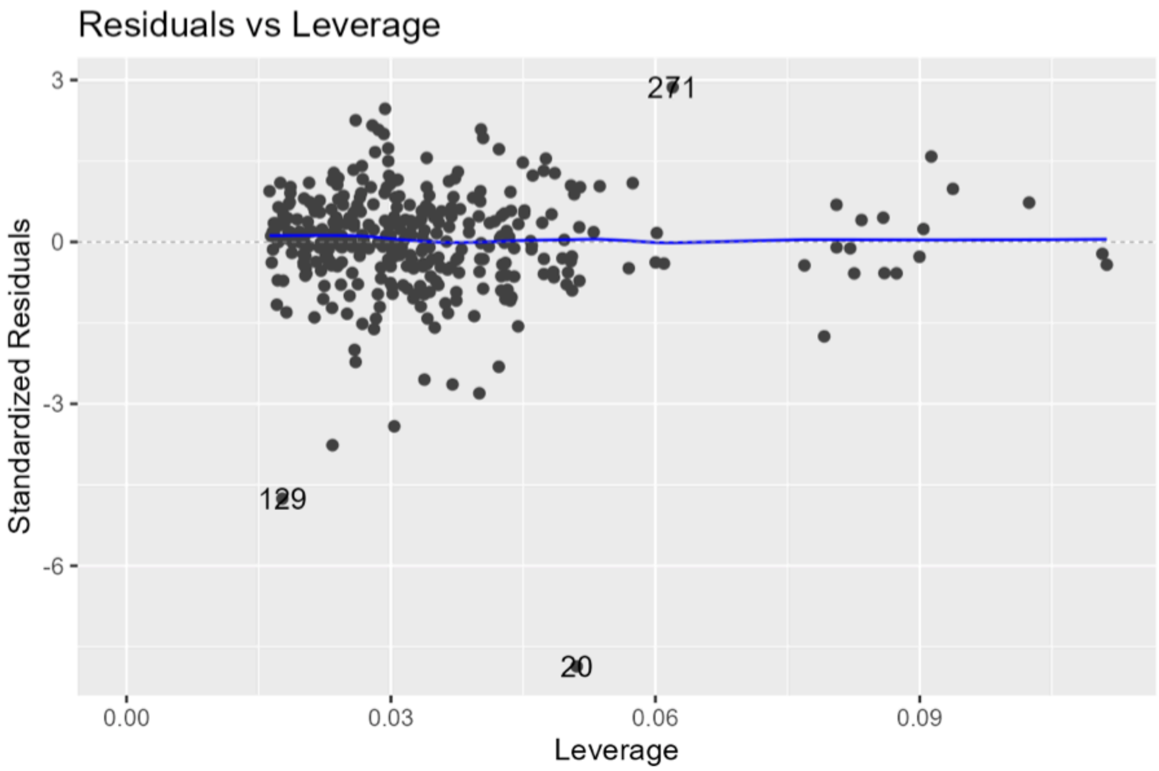

Supplementary figure 4: Diagnostics plot showing distribution of residuals in the multivariable linear regression model (figure 3) following log transformation.

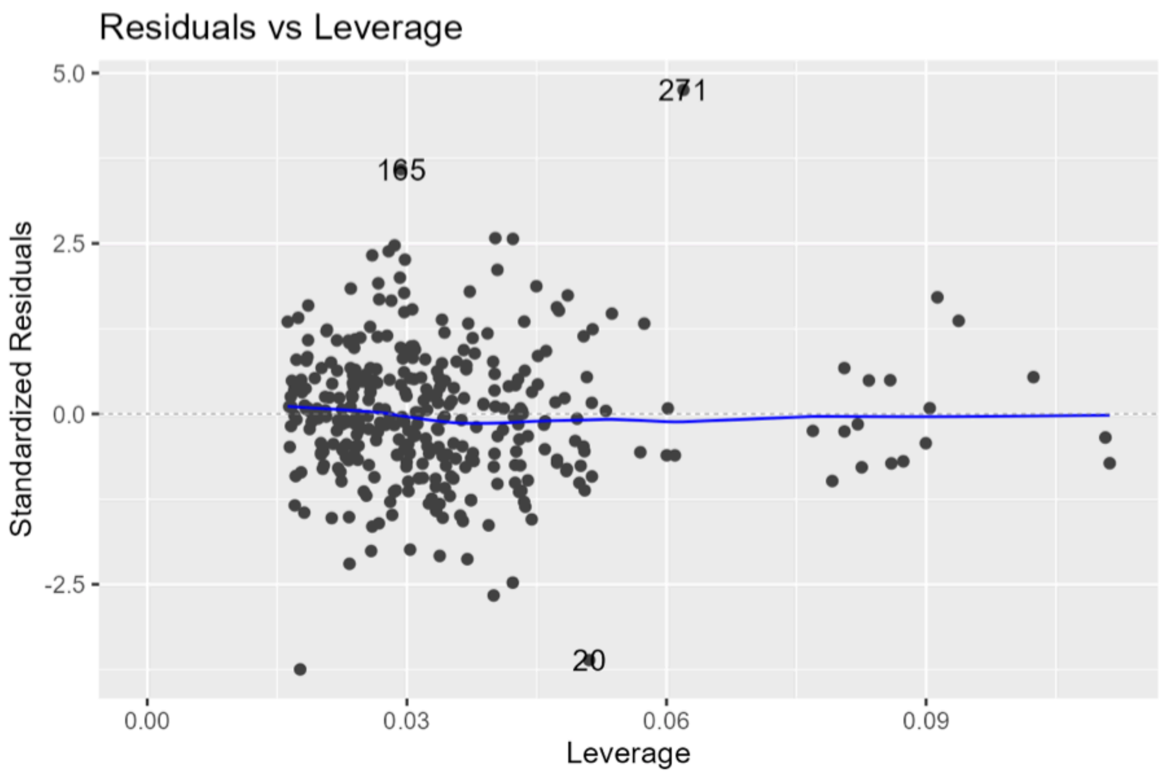

Supplementary figure 5: Diagnostics plot showing distribution of residuals in the multivariable linear regression model (figure 3) following Box Cox transformation with lambda = 0.20 (based on optimising the log-likelihood of the model).

Variable	N	Coefficient	Coefficient (95% CI)	p
Thiopurine	114/348		-0·43 (-0·77, -0·10)	0·011
Infliximab	95/348		-2·06 (-2·42, -1·70)	<0·0001
Ustekinumab	44/348		-0·47 (-1·01, 0·06)	0·084
Vedolizumab	46/348		-0·24 (-0·71, 0·23)	0·32
Tofacitinib	26/348		-0·81 (-1·36, -0·26)	0·0040
Homologous vaccination	146/348		0·16 (-0·11, 0·44)	0·24
Crohn's disease	156/348		0·13 (-0·20, 0·46)	0·45
Age (per decade)	348/348		-0·11 (-0·21, -0·01)	0·027
Non-white ethnicity	62/348		0·43 (0·08, 0·79)	0·016
Current smoker	17/348		-0·54 (-1·16, 0·08)	0·085
Prior infection	121/348		0·64 (0·36, 0·92)	<0·0001

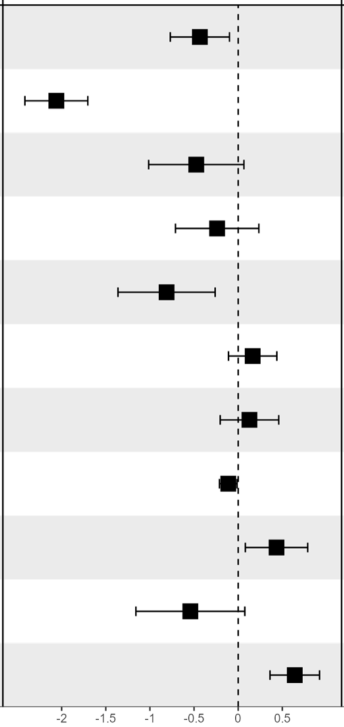

Supplementary figure 6: Sensitivity analysis using a one-parameter Box Cox transformation with lambda = 0.20 (based on optimising the log-likelihood of the model). Multivariable model showing exponentiated coefficients of linear regression models of log(anti-SARS-CoV-2 spike antibody concentration). Results are for individuals without evidence of previous SARS-CoV-2 infection. The values shown represent geometric mean ratio estimates of S1 level associated with each variable. Age was treated as a continuous variable in the analysis and its coefficient is expressed per decade.

First Name	Surname
Ijeoma	Chukwurah
Sulaimaan	Haq
Parita	Shah
Stephanie	Wilken-Smith
Anitha	Ramanathan
Mikin	Patel
Lidia	Romanczuk
Rebecca	King
Jason	Domingo
Djamila	Shamtally
Vivien	Mendoza
Joanne	Sanchez
Hannah	Stark
Bridget	Knight
Louise	Bee
Charmaine	Estember
Anna	Barnes
Darcy	Watkins
Sam	Stone
John	Kirkwood
Marian	Parkinson
Helen	Gardner-Thorpe
Kate	Covil
Lauranne	Derikx
Beatriz	Gros Alcalde
Irish	Lee
Bessie	Cipriano
Giuseppe	Ruocco
Manisha	Baden
Graham	Cooke
Katrina	Pollock
Evgenia	Kourampa
Ciro	Pasquale
Elena	Robisco-Diaz
Suhaylah	Bhatti
